# Feature-Level Analysis of a Smoking Cessation Smartphone App Based on a Positive Psychology Approach: Prospective Observational Study

**DOI:** 10.2196/38234

**Published:** 2022-07-28

**Authors:** Bettina B Hoepper, Kaitlyn R Siegel, Hannah A Carlon, Christopher W Kahler, Elyse R Park, Steven Trevor Taylor, Hazel V Simpson, Susanne S Hoeppner

**Affiliations:** 1 Recovery Research Institute Department of Psychiatry Massachusetts General Hospital Boston, MA United States; 2 Department of Psychology University of New Mexico Albuquerque, NM United States; 3 Center for Alcohol and Addiction Studies Department of Behavioral and Social Sciences Brown University School of Public Health Providence, RI United States; 4 Mongan Institute Department of Psychiatry Massachusetts General Hospital Boston, MA United States; 5 Obsessive-Compulsive Disorder and Related Disorders Program Department of Psychiatry Massachusetts General Hospital Boston, MA United States

**Keywords:** mHealth, smartphone, smartphone app, smoking, smoking cessation, nondaily smoking, positive psychology, happiness, positive affect, clinical trial, feasibility, acceptability, app usage, mobile health

## Abstract

**Background:**

Smoking cessation smartphone apps have emerged as highly accessible tools to support smoking cessation efforts. It is unknown how specific app features contribute to user engagement over time and relate to smoking outcomes.

**Objective:**

To provide a feature-level analysis of the Smiling Instead of Smoking app (version 2) and to link feature use to subsequent smoking cessation.

**Methods:**

Nondaily smokers (N=100) used the app for a period of 49 days (1 week before quitting and 6 weeks after quitting). Participants self-reported 30-day point-prevalence abstinence at the end of this period and at a 6-month follow up (the survey response rate was 94% and 89% at these points, respectively). Self-reported 30-day point prevalence abstinence rates were 40% at the end of treatment and 56% at the 6-month follow up. The app engaged users in both positive psychology content and traditional behavioral smoking cessation content. The app sent push notifications to prompt participants to complete prescribed content (ie, a “happiness exercise” every day and a “behavioral challenge” to use the app’s smoking cessation tools on 15 out of 49 days). Actions that participants took within the app were timestamped and recorded.

**Results:**

Participants used the app on 24.7 (SD 13.8) days out of the 49 prescribed days, interacting with the happiness content on more days than the smoking content (23.8, SD 13.8 days vs 17.8, SD 10.3 days; t_99_=9.28 [2-tailed]; *P*<.001). The prescribed content was frequently completed (45% of happiness exercises; 57% of behavioral challenges) and ad libitum tools were used on ≤7 days. Most participants used each ad libitum smoking cessation tool at least once, with higher use of personalized content (≥92% used “strategies,” “cigarette log,” “smoke alarms,” and “personal reasons”) than purely didactic content (79% viewed “benefits of quitting smoking”). The number of days participants used the app significantly predicted 30-day point-prevalence abstinence at the end of treatment (odds ratio [OR] 1.05, 95% CI 1.02-1.09; *P*=.002) and at the 6-month follow up (OR 1.04, 95% CI 1.008-1.07; *P*=.01). The number of days participants engaged with the happiness content significantly predicted smoking abstinence at the end of treatment (OR 1.05, 95% CI 1.02-1.08; *P*=.002) and at the 6-month follow up (OR 1.04, 95% CI 1.007-1.07; *P*=.02). This effect was not significant for the number of days participants engaged with the smoking cessation content of the app, either at the end of treatment (OR 1.04, 95% CI 0.996-1.08, *P*=.08) or at the 6-month follow up (OR 1.02, 95% CI 0.98-1.06; *P*=.29).

**Conclusions:**

Greater app usage predicted greater odds of self-reported 30-day point-prevalence abstinence at both the end of treatment and over the long term, suggesting that the app had a therapeutic benefit. Positive psychology content and prescriptive clarity may promote sustained app engagement over time.

**Trial Registration:**

ClinicalTrials.gov NCT03951766; https://clinicaltrials.gov/ct2/show/NCT03951766

## Introduction

Mobile technologies have recently emerged as highly accessible support tools for health behavior change and mental health promotion. This has been particularly true for smoking cessation, with notable increases in the use of [1,2] and referral to [3] mobile technologies designed to support quitting. User engagement with smartphone apps, however, represents a critical challenge. It has been estimated that less than 5% of apps continue to be used 15 to 30 days after the initial app download [4]. Note that to our knowledge, a standardized operational definition of app engagement has not yet been established [5]; throughout this paper, we use the term “app engagement” to denote user behavior, that is, an app user interacting with the app’s user interface.

App engagement is important for several reasons. First, it is through interaction with an app that app users engage in therapeutic activities. Such engagement can take different forms. An app may present information, provide a tool or behavior change strategy, assign homework, or prompt the app user to take specific actions (eg, call a clinician) [1]. It can be assumed that the more app users interact with the app, the more they will engage in thoughts, feelings, and actions that are believed to be beneficial to their smoking cessation goal. This logic is consistent with findings across the eHealth literature demonstrating that greater engagement with eHealth tools (eg, websites) is associated with more favorable outcomes related to smoking cessation [6-8].

A critically useful feature of smartphone apps is their potential utility in providing sustained support over time. Several studies suggest that extending behavioral support helps smokers remain abstinent in the long term, with longer treatments lasting 8 to 12 weeks [9-11]. If longer treatment is better, the next question is what app users should be doing within a smoking cessation app. The vast majority of publicly available smoking cessation apps focus on simple tools: calculators to track money saved and health benefits accrued or calendars to track the days until or since the chosen quit day [1]. The apps provided on Smokefree.gov, a recommended mHealth referral site for treating smokers in health care settings [3], are more sophisticated. They offer a variety of tools and trackers (eg, time-based and GPS-based reminders to abstain from smoking at high-risk times or in high-risk locations and daily tips and milestone achievement badges) while also providing tailored feedback for overcoming urges to smoke due to cravings and mood states. These apps are able to capture the initial engagement of a very large number of smokers (there are over 25,000 and 13,000 users to date for QuitGuide and quitSTART, respectively) [12]. It is unclear, however, to what extent and how these apps sustain engagement over time.

A new generation of smoking cessation apps is emerging. In such apps, there are additional ways that smoking cessation is supported. The nature of these approaches varies widely from app to app, including contingency management [13,14], motion-sensor detection of smoking [15], carbon monoxide level monitoring [16], using gaming to engage smokers in skills practice [17] or other activities promoting smoking cessation [18], promoting nicotine replacement therapy adherence [19], prescribing bouts of physical activity [20], mindfulness training [21], and acceptance and commitment therapy (ACT) [22,23]. Most of these apps are in the early stages of development, and study protocols have been published, but the studies are ongoing. A handful of pilot feasibility studies exist, only a few of which offer insight into app usage over time. App usage ranged from 7 days for a smartphone app using a gamification approach to smoking cessation [18], to 34 and 32 days, respectively, in a pilot randomized control trial that compared an ACT-based app to the National Cancer Institute (NCI)’s app QuitGuide [22]. The largest randomized trial to date reported 24 days of app use for the app iCanQuit, an ACT-based smoking cessation app, compared to 7 days of use for an earlier version of NCI’s QuitGuide, a version that was largely text-based and did not include trackers or tools [23].

In this paper, we present a feature-level analysis of app usage over time of the Smiling Instead of Smoking (SiS) app. To our knowledge, this is only the second research project that links feature-level app use to subsequent smoking cessation. The first such project provided a feature-level analysis of the app SmartQuit [24], an early version of iCanQuit [23]. The feature-level analysis of SmartQuit [24] indicated that the use of 3 features was prospectively linked to smoking cessation at follow up; 2 of these features were ACT focused (ie, tracking ACT skills practice and tracking the practice of letting urges pass); the remaining feature was a traditional US Clinical Practice Guidelines (USCPG) feature (ie, viewing the quit plan) [24]. In that analysis, the team noted little overlap between the popularity of the app’s features and their subsequent link to smoking cessation success.

The app we are examining in this paper, the SiS app, was developed specifically for nondaily smokers [25-27]. Nondaily smoking is a widespread, increasingly prevalent pattern of smoking. Currently, 24.3% of all adult smokers smoke on a nondaily basis [28], which constitutes a 27% increase over the past decade [29]. Despite such prevalence, particularly in ethnic minority groups [30-34] and vulnerable populations, such as persons with mental health and substance use challenges [35], behavioral and pharmacological recommendations for nondaily smoking remain unaddressed in clinical practice guidelines [36]. Nicotine replacement therapies have been tried but so far have failed to show efficacy in achieving smoking abstinence in nondaily smokers [37,38], in line with a lack of interest among nondaily smokers in pharmacotherapy for smoking cessation [39,40]. Nondaily smokers are, however, highly motivated to quit smoking. Compared to daily smokers, they have greater current intentions to quit smoking [31,41,42] and more recent and planned cessation efforts [42-45]. These factors point to the utility of behavioral support, which can be delivered effectively via smartphone technology.

The therapeutic goal of the SiS app is to maintain positive affect while smokers undergo a quit attempt. Positive affect often decreases during a quit attempt [46], as smokers struggle with cravings and adjusting to a smoke-free life. Indeed, recently, a decrease in positive affect has been suggested as a new symptom of tobacco withdrawal, based on data from 24 trials involving 2054 participants showing a medium effect size (Cohen *d*=–0.40) for an overall decrease in positive affect [47]. Maintaining positive affect during this time, however, may be especially beneficial, because greater positive affect is associated with increased self-efficacy to quit smoking [48], decreased desire to smoke [49,50], and greater readiness to process self-relevant health information [51], all of which are constructs highlighted in dominant health behavior theories as causal agents in successful behavioral change [52-56].

Smoking cessation apps can deliver a wide array of content, but users must engage with the information to benefit. The SiS app engages users in short, daily exercises to boost their positive affect. This boost in positive affect is intended to increase their readiness to engage in the smoking cessation materials provided in the app [51]. Moreover, completing happiness-boosting exercises is intrinsically rewarding, in and of itself. Having rewarding experiences while using the app may, in turn, entice app users to return to the app on subsequent days. This dynamic is evident across a diversity of settings, and positive psychology interventions have been found to be highly appealing to patients [57], resulting in better treatment adherence [58,59] and engagement [60].

In the initial study of version 1 of the SiS app (N=30), the “happiness exercises” appeared to be a driving factor in app engagement [26]. In this paper, we are examining app usage of participants in a larger trial, using version 2 of this app. The overall app usage was quite high, with smokers using the SiS app for an average of 24 days within the prescribed period of 49 days [27]. Using these data, our goals were to (1) describe how nondaily smokers used the app and (2) test if app usage patterns (ie, any app use, use of happiness content, and use of smoking content) during the prescribed treatment period predicted smoking abstinence (ie, self-reported 30-day point-prevalence abstinence [PPA]) at the end of treatment and at a 6-month postquit follow up.

## Methods

### Participants

In this secondary data analysis, we examined app usage data from 100 adult nondaily smokers participating in a single-arm study. The eligibility criteria for the study were as follows: age at least 18 years, nondaily smoking habit (ie, smoking at least weekly but no more than 25 out of the past 30 days), smartphone ownership (Android or iPhone only), willingness to make a quit attempt as part of the study, willingness to name friends and family members who could help study staff with updating contact information for follow-up assessments, and fluency in the English language. Note that our operational definition of nondaily smokers was designed to include the majority of nondaily smokers while targeting nondaily smokers who would routinely engage in nondaily smoking and thus might benefit from an app providing continuous support, and nondaily smokers who were not too close to being daily smokers. We have kept this operational definition consistent across our studies on nondaily smokers [26,40]. Nationally representative data at the time suggested that it would include 72% of nondaily smokers [31]. Participants who completed a baseline survey and who were successfully onboarded to the app via phone were included in this secondary data analysis. The average age of participants was 35.9 (SD 11.4) years. More than half (61/100, 61%) were female, and the majority were white (75/100, 75%) and employed (63/100, 63%), either full-time (44/100, 44%) or part-time (19/100, 19%). Thirty-eight participants (38/100, 38%) had a college degree or higher. Most had previously smoked daily (70/100, 70%). Typically, approximately half of nondaily smokers are former daily smokers [61-63]. Most participants had previously tried to quit smoking (77/100, 77%), a rate that was slightly higher than that reported by nondaily smokers in the 2000 National Health Interview Survey, in which 65% reported having tried to quit [42]. Participants smoked on average on 14.7 (SD 4.6) days out of the past 30 days and smoked 4.6 (SD 3.3) cigarettes per smoking day. Half had tried e-cigarettes (57/100, 57%).

### Procedures

Recruitment occurred between June and November 2019. Participants were recruited nationwide using online resources. After screening, participants completed an onboarding phone call with study staff, during which they were guided through downloading, installing, and using the app. This onboarding phone call marked the beginning of the treatment period: participants were instructed to use the app for the subsequent 49 days. The onboarding call was scheduled to occur 1 week prior to the participants’ chosen quit day, so that the prescribed app use period covered 1 week before and 6 weeks after the initial quit day (participants could reschedule their quit day within the app). App usage for all participants was recorded by the app. Participants also completed online surveys 2, 6, 12, and 24 weeks after the initial quit day, with response rates of 96/100 (96%), 96/100 (96%), 94/100 (94%), and 89/100 (89%), respectively.

### Ethics Approval

The study procedures were approved by the Mass General Brigham Institutional Review Board (2018P002699) and are detailed elsewhere [27]. The trial has been registered on ClinicalTrials.gov (NCT03951766).

### Outcomes

#### App Utilization

Actions that participants took within the app were timestamped by the app and recorded on a secure server. From these data, we coded the number of days participants used certain features of the app and the percentage of participants who used that feature at least once after onboarding.

#### Smoking Cessation

In online surveys, participants were asked to indicate their smoking status using the following options: “I smoke daily,” “I smoke nondaily (and have smoked in the past 7 days),” “I smoke nondaily (but have NOT smoked in the past 7 days),” and “I do not smoke at all.” Participants who reported not smoking at all were then asked if they had been completely abstinent since their originally chosen quit day, during the past 7 days, and during the past 30 days. From this, we coded 30-day PPA at the end of treatment and at the 6-month follow up.

#### Description of the SiS App

**Figure 1:**
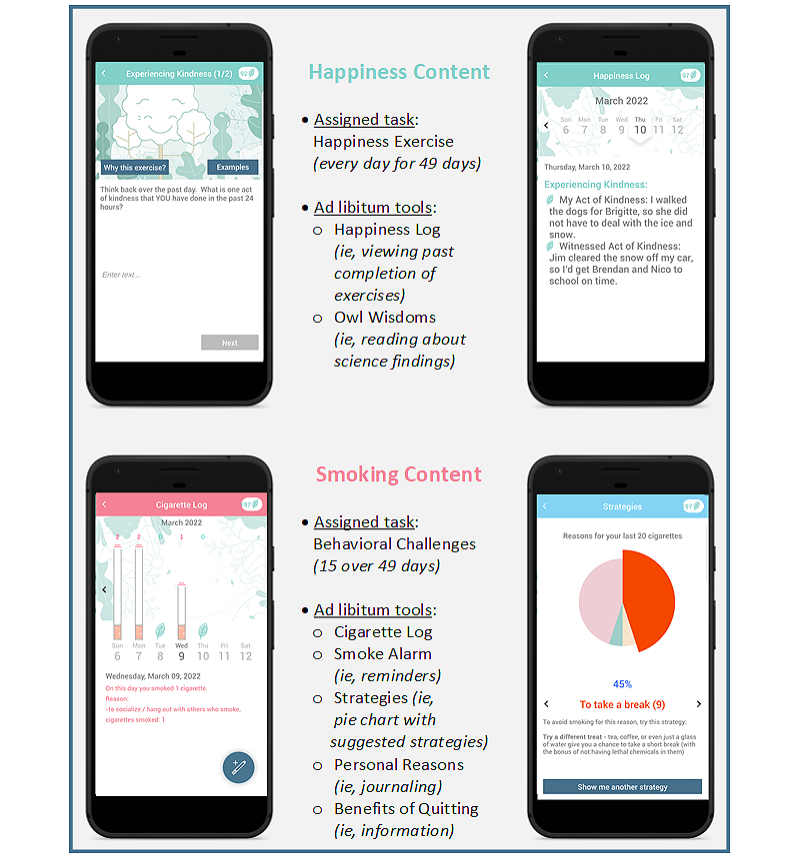
Features of the Smiling instead of Smoking 2 app.

Version 2 of the SiS app (Figure 1) engaged app users in both positive psychology content designed to maintain their positive affect and traditional behavioral smoking cessation content to guide their quit attempt. During onboarding, study staff walked participants through the app and how to use it. They started with the happiness content, including showing the participants the specific buttons that explained the positive psychology framework used by the app (ie, the buttons labeled “why happiness” and “why this exercise”). These buttons were prominently displayed when engaging in the positive psychology content of the app and provided text explaining why app users were asked to complete happiness exercises in order to support their smoking cessation efforts. Study staff then moved on to “behavioral challenges,” and used these as an organizing structure to guide participants through the smoking tools.

To elicit positive affect, participants were asked to complete a happiness exercise each day. Each day, the app chose 1 of 5 happiness exercises (Multimedia Appendix 1) to be completed that day. To complete the exercise, participants had to enter text into the app (eg, to describe good things that had happened to them or to describe something they had savored). These 5 exercises had been tested previously in an online survey that randomized survey takers into completing 1 of these exercises or 1 of 2 control exercises, which showed that these happiness exercises increased in-the-moment happiness [64]. Optionally, app users could review their past entries in the “happiness log,” and could use the feature called “owl wisdoms” to read about scientific findings that showcase the utility of engaging in happiness-enhancing activities.

For smoking content, participants were asked to complete temporally appropriate “behavioral challenges” every 3 to 4 days (on 15 of 49 days). These behavioral challenges were anchored on the participant’s quit day, which they specified in the app upon app installation. Participants could reset the quit day at any point, causing the app to adjust the schedule of the behavioral challenges accordingly. These behavioral challenges prompted users to use the smoking cessation tools provided within the app, in the order recommended by the NCI’s “Clearing the Air” brochure [65]. The tools included a cigarette log to log smoked cigarettes, a strategy guide, which provided a pie chart of users’ smoking triggers and suggested strategies for them, an alarm feature that let users set reminders to stay smoke free at upcoming times and events, a journal function to enter personal reasons for quitting smoking, and an informational section where the benefits of quitting smoking were presented. After the first month, behavioral challenges also directed participants to use the app’s ad libitum happiness tools (ie, the happiness log and “owl wisdoms”).

In total, app use entailed both prescribed (ie, happiness exercises and behavioral challenges) and ad libitum app activities that pertained to either happiness or smoking cessation (Figure 1). During the onboarding call, study staff set clear expectations that the participants should complete the happiness exercise every day for 49 days (ie, during the treatment period) and optionally thereafter and that they should complete every behavioral challenge. The app sent push notifications to prompt participants to complete the prescribed content. For the happiness exercise, the push notification was either sent at 10 AM to announce the exercise, at a random time between 12 PM and 2 PM to remind them to complete the exercise, or at 7 PM (if the exercise was still incomplete). For behavioral challenges, the push notification was always sent at 10 AM.

#### Analyses

To describe app use during the prescribed 49-day app use period, we calculated the average number of days on which participants used the app overall and specific functions within the app. We also calculated the percentage of participants who used specific functions of the app at least once after onboarding and the percentage of times the prescribed activities (ie, happiness exercises and behavioral challenges) were completed. To test if participants seemed to prefer one happiness exercise over another, we calculated the number of times the participants completed each of the 5 exercises and then used a hierarchical linear model to test if the categorical variable denoting each exercise significantly predicted this number. Observations were modeled as nested within persons.

To test if app usage during the prescribed treatment period predicted smoking abstinence, we used a series of univariate logistic regressions, where self-reported 30-day PPA was the dependent variable (with 1 indicating “abstinent” and 0 indicating “not abstinent”) and app usage was the univariate predictor. We examined 3 different summaries of app usage: the overall number of days the app was used, the number of days the happiness content was used, and the number of days the smoking cessation content was used. Based on the app usage pattern observed in the first SiS study [26], we expected the correlation of the number of days the app was used and the number of days the happiness content was engaged with to be very high. We calculated them separately, however, to create conceptual clarity in our prediction of smoking cessation. We fit the same models for smoking abstinence at the end of treatment (ie, 6 weeks after the initially chosen quit day) and at the end of follow up (ie, 6 months after the initially chosen quit day). Participants were assumed to be smoking if they did not complete the surveys (there were 4% and 11% nonresponse rates at weeks 6 and 24, respectively). The logistic regression results are presented with odds ratios (ORs) and the Wald 95% CI, as well as the *C* statistic (an indicator of correct classification). All analyses were completed in SAS 9.4 for Windows (SAS Institute).

## Results

### App Usage

**Figure 2:**
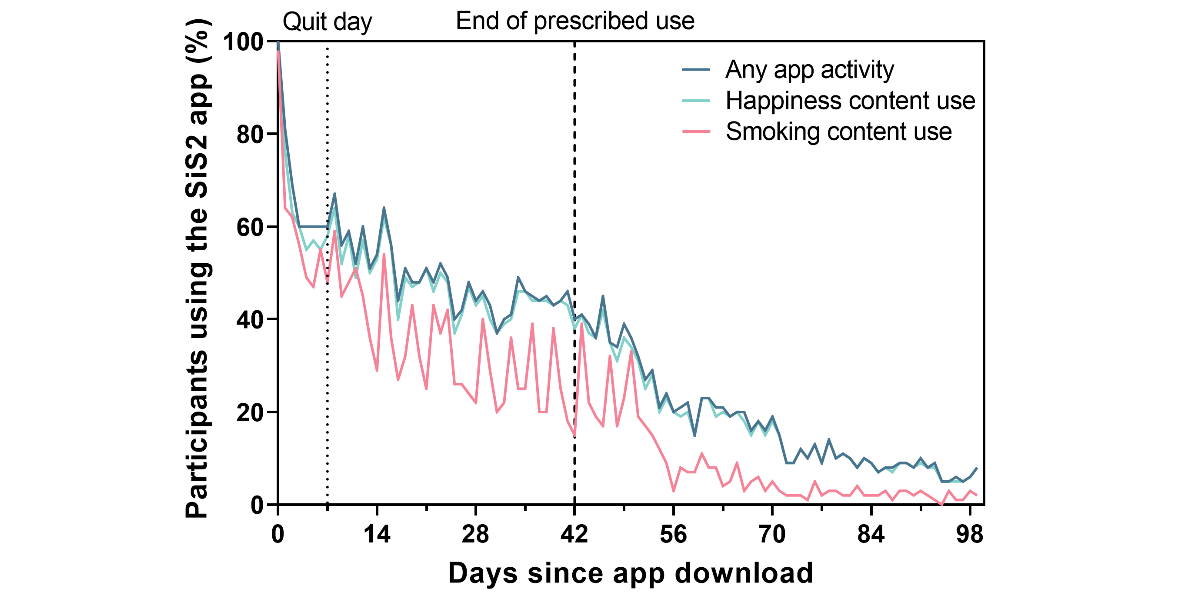
App usage over time. SiS2: Smiling Instead of Smoking, version 2.

Participants used the SiS app an average of 24.1 (SD 14.1) days out of the 49 prescribed days (Figure 2). Overall, they interacted with the happiness content on more days than the smoking-related content (23.2 days, SD 14.1, vs 16.7 days, SD 10.3; t_99_=9.47 [2-tailed]; *P*<.001). Participants completed the behavioral challenges more consistently than the happiness exercises, with participants completing 56.6% (SD 28.1%) of the behavioral challenges, on average, compared to 44.8% (SD 28.8%) of the happiness exercises (t_99_=7.44; *P*<.001). The completion rate of the happiness exercises differed by exercise type (*F*_4,396_=2.82; *P*=.03). Tukey adjusted posthoc pairwise comparisons showed that participants completed the “rose, thorn, and bud” exercise more often than the “savoring” exercise (4.6 days, SD 3.1, vs 4.1 days, SD 2.8; *P*=.02). The completion rates of the other 3 exercise types (“3 good things”: 4.5 days, SD 3.0, “experiencing kindness”: 4.4 days, SD 3.1, and “reliving happy moments”: 4.4 days, SD 3.0) were intermediate to the “rose, thorn, and bud” and “savoring” exercises and did not differ from any other exercise type.

Ad libitum tools were used relatively sparingly. The behavioral challenges appeared to have been successful in initially engaging participants with specific tools, as indicated by the high percentage of participants using each tool at least once after the onboarding day (Table 1). For example, 93 participants (93%) used the “personal reasons” tool, and 93 participants (93%) used “smoke alarms” at least once after onboarding. The number of days on which participants used these smoking cessation tools, however, was relatively low. Of these tools, the “cigarette log” was used the most (average 7.2 days), and the “benefits of quitting” the least (average 2.5 days). The happiness-focused ad libitum tools were similarly infrequently used. The exception was the happiness log, which was viewed on 22.1 of 49 days; however, it should be noted that upon completion of the assigned happiness exercises, participants automatically landed on the happiness log.

**Table 1:** Description of app use during the prescribed app use period (ie, 49 days).

App use		Days used of possible 49, mean (SD)	Participants (N=100) with at least one day of use after onboarding, n (%)
**Overall**			
	Any use of the app	24.1 (14.1)	99 (99)
	Any happiness content	23.2 (14.1)	98 (98)
	Any smoking content	16.7 (10.3)	98 (98)
**Assigned tasks**			
	“Happiness exercises” completed	22.0 (14.1)	96 (96)
	“Behavioral challenges” completed	8.5 (4.2)	100 (100)
**Ad libitum tasks**			
	“Happiness log” viewed	22.1 (14.1)	96 (96)
	“Owl wisdoms” viewed	3.6 (3.3)	78 (78)
	“Happiness information” viewed	2.0 (3.2)	60 (60)
	“Cigarette log” viewed/edited	7.2 (6.5)	96 (96)
	“Smoke alarms” viewed/edited	4.6 (3.0)	93 (93)
	“Strategies” viewed/edited	4.1 (3.3)	94 (94)
	“Personal reasons” viewed/edited	3.5 (3.2)	92 (92)
	“Benefits of quitting” viewed	2.5 (2.3)	79 (79)

### Relationship of App Usage to Smoking Abstinence

**Figure 3:**
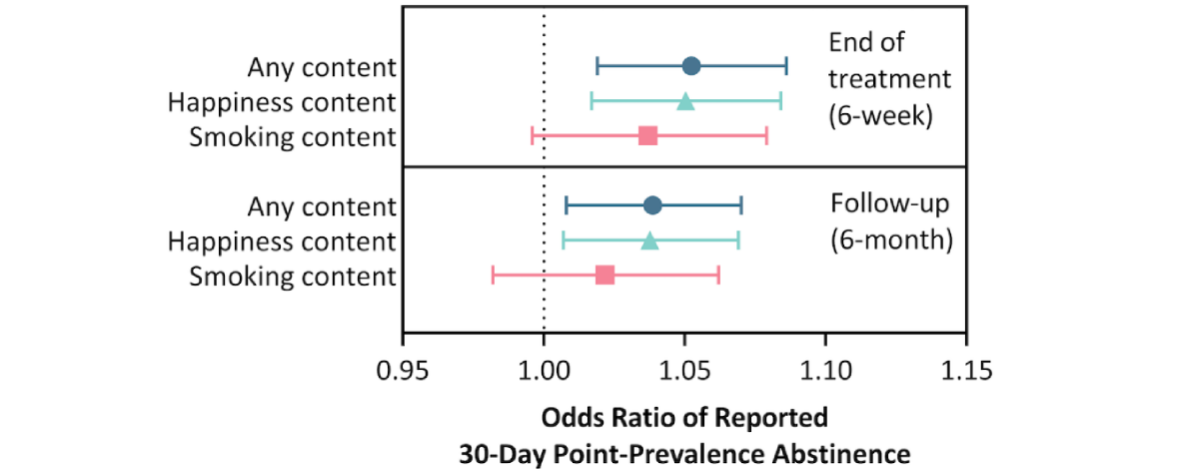
Odds ratio of app usage predicting self-reported 30-day point prevalence abstinence. The odds ratio is based on a single-day increase in app usage of the indicated content (ie, "any content," "happiness content," "or smoking cessation content").

As illustrated in Figure 3, overall, the number of days with any use of the app significantly predicted smoking abstinence at 6 weeks (1 more day of use: OR 1.052, 95% CI 1.019-1.086; *P*=.002; *C*=.69) and 6 months postquitting (1 more day of use: OR 1.038, 95% CI 1.008-1.070; *P*=.014; *C*=.65). The number of days participants engaged with the SiS 2 app’s happiness content significantly predicted smoking abstinence at the end of treatment (1 more day of use: OR 1.050, 95% CI 1.017-1.084; *P*=.002; *C*=.69) and at 6-month follow up (1 more day of use: OR 1.037, 95% CI 1.007-1.069; *P*=.016; *C*=.65). This effect was not significant for the number of days participants engaged with the smoking cessation content of the SiS 2 app, at either the end of treatment (1 more day of use: OR 1.036, 95% CI 0.996-1.079; *P*=.08; *C*=.64) or at the 6-month follow up (1 more day of use: OR 1.021, 95% CI 0.982-1.062; *P*=.29; *C*=.59). The correlation between these 3 app usage indices was high, especially between any content and happiness content (*r*=0.995), but only somewhat lower for smoking content with any content (*r*=0.89) and happiness content (*r*=0.88).

## Discussion

### Key Findings

This secondary data analysis of participants enrolled in a smartphone app–based smoking cessation study provided insight into how feature-level app usage behaviors relate to smoking cessation outcomes. The app under study, the SiS 2 app, is part of an emerging generation of smartphone apps that offer a treatment framework beyond standard USCPG content. In our feature-level analysis of the SiS 2 app, we found that overall greater app usage predicted higher chances of subsequent smoking cessation. This finding is in line with the feature-level analysis of the app SmartQuit [24], which showed that greater app use was positively related to subsequent smoking cessation. It suggests that there was a potential therapeutic effect of engaging with the SiS 2 app, though a causal relationship could not be established in this observational study.

Divergent from SmartQuit findings, for SiS 2 the popularity of the app’s features aligned with smoking cessation success, where greater usage of the happiness components of the app predicted greater chances of 30-day PPA at both the end of treatment and at the 6-month follow up. This finding suggests that the positive psychology components of SiS 2 are an important factor in supporting smoking abstinence. This is in line with findings from in-person treatment studies that indicate the value of positive psychology in smoking cessation [66,67]. Our findings here suggest this value may extend to the smartphone app environment. Particularly noteworthy is the high level of engagement with the SiS 2 app (ie, sustained use over 49 days, with multiple uses per week), largely driven by the positive psychology content. In mHealth research, touch point frequency is an emerging area of investigation, with some apps focusing solely on reminding app users not to smoke at key timepoints [15], as simple reminders can be powerful tools in smoking cessation [68]. The high touch point frequency observed for the SiS 2 app speaks to its ability to remain present in smokers’ minds as they navigate smoking cessation.

### Prescriptive Clarity

Our results highlighted a rather stark difference in the completion rates of assigned versus ad libitum tasks. Happiness exercises were completed on 45% of days, and 57% of assigned behavioral challenges were completed. These completion rates are in line with the completion rates reported for a mindfulness smoking cessation app for adolescents; participants completed 13 of 22 (61%) of the assigned mindfulness modules [69]. The ad libitum tools, on the other hand, were sparsely used: they were used on only 7 days for the most popular ad libitum tool. This finding suggests that prescriptive clarity may be of critical importance in driving app usage, and therefore in achieving an app’s therapeutic effect. In the SiS 2 app, there was prescriptive clarity: clear expectations were set about treatment length (ie, 49 days), and which specific actions to complete (ie, daily happiness exercises and 15 behavioral challenges). These expectations were reinforced with proactive push notifications. The iCanQuit app [70] also had prescriptive clarity: a set number of modules were required to be completed. Both apps had high app engagement over time. By contrast, the NCI app QuitGuide lacks prescriptive clarity. Many potentially useful tools are offered by QuitGuide, but it is not clear which tools to use, when, and for how long. Future research that experimentally tests whether prescribed content is more engaging would be useful to inform the development of health behavior apps.

To date, text messaging has shown greater smoking cessation benefits than smartphone apps [71], potentially in part due to prescriptive clarity. Text-messaging interventions have prescriptive clarity (ie, there is a set number of days in the program; information is provided on specific days, in proactive, succinct fashion; and actions to be taken are clearly spelled out), while many apps do not [1]. Our data show that participants are willing to complete assigned tasks much more than use ad libitum tools. Data from a randomized trial conducted in the United Kingdom show that assigning daily tasks within a smoking cessation app versus offering the same content without the specific daily tasks led to improved smoking cessation rates [72]. Combined, these findings lead us to believe that prescriptive clarity is a critically important feature in the development of smartphone apps targeting smoking cessation.

### Long-term Engagement

Smartphone app technology has the potential to provide ongoing support for smoking cessation over long periods of time. To date, this potential has been largely unexplored, including in our own work. To our knowledge, few studies have examined app engagement; these studies have focused on factors contributing to initial app use [73] or have tested the value of push notifications in enhancing engagement [74]. These studies have not provided insight into the content features and app parameters that promote long-term engagement. In-person smoking cessation interventions typically provide 8 to 12 weeks of support. The SiS 2 app provided assigned tasks for 49 days and continued use of the app’s ad libitum tools as needed, a treatment length in line with the support offered via the NCI’s Smokefree text-messaging interventions, and similar to the UK Smoke Free app, which provides assigned daily tasks for 31 days [72]. This treatment length roughly covers the time from preparation to action according to the transtheoretical model of change and does not address maintenance [55]. In fact, originally, the SiS app spanned only 21 days [26], a length that was specifically chosen to provide support during the acute “cessation” phase [75] of the process of smoking cessation. Based on user feedback, we increased treatment length to 49 days in version 2. Our app usage data demonstrate that this increased treatment length was well tolerated, opening the door to potentially further increasing treatment length to provide support during the maintenance phase of smoking cessation. To date, however, very little research exists to guide the intervention content of smartphone apps to support sustained user engagement and long-term abstinence from smoking.

### Limitations

This secondary data analysis was based on a single-arm trial, and therefore causal inferences about the observed effects cannot be drawn. Our analyses were exploratory. They hint at the value of positive psychology to engage app users in smoking cessation over time and the value of prescriptive clarity, but we did not design this study, nor the SiS 2 app, to address these questions. In considering these effects, it should be kept in mind that the participants were asked to complete happiness content on a more frequent basis than smoking cessation content (ie, daily vs every 3 to 4 days) and that the smoking cessation tools had a shelf-life (eg, “smoke alarms” became less useful as cravings diminished; participants could log cigarettes, but not smoke-free days). In terms of generalizability, it should be noted that the SiS 2 study used an interactive onboarding procedure via phone. While this is in line with warm handoff models for smoking cessation [76,77], app usage patterns within the context of a clinical trial are typically higher than real-life app use [78].

### Conclusions

In the SiS 2 app, greater app usage predicted greater chances of self-reporting 30-day PPA at both the end of treatment and at a 6-month follow up. This finding strengthens the rationale for testing this app in a randomized trial. Feature-level analysis of app usage patterns suggests that positive psychology content and prescriptive clarity may promote app engagement.

## Appendix

Multimedia Appendix 1 Happiness Activities.

## Abbreviations

ACT: acceptance and commitment therapy
NCI: National Cancer Institute
OR: odds ratio
PPA: point-prevalence abstinence
SiS: Smiling instead of Smoking
USCPG: US Clinical Practice Guidelines
