# Supplementary material for: Feature-Level Analysis of a Smoking Cessation Smartphone App Based on a Positive Psychology Approach: Prospective Observational Study
Source: JMIR Form Res. 2022 Jul 28;6(7):e38234. doi: 10.2196/38234 (PMC9377446; doi:10.2196/38234)
Supplement: Multimedia Appendix 1 [file formative_v6i7e38234_app1.docx]

**Happiness Activities**

Version 2 of the Smiling instead of Smoking (SiS) app uses five happiness exercises, with one of these five exercises chosen at random each day by the app to be completed that day. To get to the day’s happiness exercise, app users open the app, click on “Happiness”, which brings them to the “Happiness Exercises” screen, and then click on “Today’s Happiness Exercise”. Then the day’s exercise is shown (see example below). App users enter text in the provided text box to complete the exercise.

All five happiness exercises are formatted in the same way. They each provide a button to learn more about “why this exercise”, and another button, “Examples”, that provides examples of how to complete this exercise.

The table below provides the names of the exercises, and the instructions participants read before entering text to complete the exercise:

| **Name of Happiness Exercise** | **Instructions to App Users** |
| --- | --- |
| 3 Good Things | Think back over the past day. What are three good things that you have experienced in the past 24 hours? |
| Experiencing Kindness | Think back over the past day. What is one act of kindness that YOU have done in the past 24 hours? What is one act of kindness that you have seen SOMEONE ELSE do in the past 24 hours? |
| Savoring | Please describe an experience that you have savored in the past 24 hours. By ‘savored’ we mean a positive experience that you have noticed and appreciated as being a wonderful moment. These experiences can be quite simple, everyday kind of moments. If you didn’t actively savor an experience in the past 24 hours, please describe an experience you will likely have in the next 24 hours that you could savor. |
| Rose, Thorn, and Bud | Think back over the past day. What was a highlight of your day (your rose), a challenge of your day (your thorn), and something you look forward to in the next 24 hours (your bud)? |
| Reliving Happy Moments | Take a minute to browse through the pictures you have saved on your smartphone, on your computer, or have lying around. Find one that brings back a happy memory. What is happening in that picture? |
